# “I Became the Messenger Between the Hospitals”: A Study on the Journeys of People With Cancer Using the Critical Incident Technique

**DOI:** 10.1111/hex.70211

**Published:** 2025-03-10

**Authors:** Ragnhild Halvorsrud, Line Melby, Kristine Gjermestad, Binyam Bogale, Ingrid Konstanse Ledel Solem

**Affiliations:** ^1^ SINTEF Digital Oslo Norway; ^2^ SINTEF Digital Trondheim Norway; ^3^ Norwegian University of Science and Technology Ålesund Norway

**Keywords:** cancer patient journey, critical incident technique, healthcare services, organisation of care, patient experience

## Abstract

**Introduction:**

Patients with serious conditions face complex, long‐lasting patient journeys involving multiple healthcare providers. Research shows that these journeys are frequently perceived as fragmented, with significant challenges in communication and information flow. However, there is limited knowledge about the organisational and informational aspects linked to good and poor experiences. This study investigated critical factors in cancer journeys, focusing on communication and the informational and organisational elements shaping patient experiences.

**Methods:**

The critical incident technique was used to identify positive and negative factors in cancer patient journeys. People with cancer and their next‐of‐kin were recruited through Norway's national cancer organisation. Patient episodes were collected from 41 participants via digital workshops combined with questionnaires and supplemented by in‐depth interviews. Critical incidents were extracted using specific analytical criteria.

**Results:**

A total of 187 critical incidents were identified, including 81 positive and 106 negative. Content analysis revealed 12 categories of incidents. Positive incidents were linked to effective communication, timely information, and well‐coordinated care, particularly through cancer pathways. Negative incidents often involved communication delays, insensitive information delivery, and poor coordination among healthcare providers. Notably, around 40% of the negative incidents stemmed from fragmented health services or a lack of progress, often forcing patients to act as messengers.

**Conclusion:**

By examining critical experiences, this study highlights key areas for improving cancer care. Timely information and clinical empathy when delivering sensitive diagnoses are essential. Healthcare providers must coordinate services more effectively to prevent patients from intervening to ensure care progress.

**Patient or Public Contribution:**

Patients' stories formed the core data. The public contributed to recruitment, while patient feedback informed the workshop design.

## 1 Introduction

Cancer care has improved remarkably in recent decades [1], with advancements in diagnostics and therapeutics enhancing survivorship [2]. However, cancer remains a global health problem, with prevalence expected to rise [3, 4, 5]. Cancer diagnostics, treatment, and follow‐up involve specialised expertise and complex collaboration across primary and specialist care [6]. For patients, this complexity translates into interactions with numerous healthcare providers (HCPs) and organisations throughout their journey [7]. Poor information and service coordination can lead to negative patient experiences and compromised quality of life [8]. Effective communication and well‐organised care are pivotal for fostering collaboration among providers. As cancer survivorship increases, patient journeys become longer and more complex, heightening the risk of fragmented care where knowledge of the illness, history, and preferences is dispersed across services [6]. Improved survivorship, increasing cancer incidence, and the inherent complexity of the disease present further challenges to care delivery from multifaceted angles [7, 9, 10].

Global efforts to optimise cancer care have focused on streamlining care organisation [6], reducing treatment variations and delays [11], and enhancing patient and next‐of‐kin experiences [7, 12, 13]. In Norway, cancer patient pathways (CPPs) were introduced in 2015 [14] to assign care coordinators and standardise communication. Although evaluations show reduced waiting times [15], results vary by age and residence [16]. Studies highlight that well‐organised, respectful, responsive, and effectively communicated care improves patient satisfaction [7, 17]. Despite improvements, patients still need more information, and CPPs must better align with patient expectations [18]. As these needs evolve, understanding the perspectives of patients and families regarding their care experience remains essential.

With the growing emphasis on patients' experiences, particularly following the rise of person‐centred care [19], evaluating quality of care (QoC) from multiple perspectives [9, 20] has become increasingly important. Traditional QoC measurements, such as patient‐reported outcome measures (PROMs) and experience measures (PREMs), are widely used in cancer care to identify positive and negative experiences [19, 21] and are increasingly integrated into healthcare systems [22]. However, these methods often provide only a snapshot of the patient experience. In contrast, the critical incident technique (CIT) offers a more comprehensive approach by capturing critical issues across the patient journey [23]. From behavioural science, we know that moments of high emotional intensity are more easily remembered [24], making CIT well‐suited for analyzing impactful experiences. Originally developed in service research [25], CIT is now widely applied in nursing and health service research [26] to gain insights into key experiences from both providers’ [27] and patients' perspectives [28]. By considering the broader context, CIT provides deeper insights into the factors that most influence patient experiences. Its retrospective nature focuses on impactful events, offering a structured approach to identifying and assessing critical incidents [26].

This study aims to identify positive and negative critical incidents related to communication, information, and care coordination throughout cancer patient journeys. It focuses on the patient perspective, extracting succinct incidents from patient‐reported episodes based on strict criteria. Incidents are analyzed through a semiotic communication model, emphasising the actors involved, communication channels, the conveyed message, and its context. Regarding terminology, “patient journey” refers to the patient‐facing process of receiving healthcare services, while the term “healthcare” refers to the complex, multi‐actor system.

## 2 Methods and Materials

### 2.1 Study Design and Participants

Following the COVID‐19 pandemic, data collection was conducted digitally to enable nationwide participation and minimise travel and exposure risks for vulnerable individuals. Online workshops were conducted [29], with small‐group digital breakout rooms focusing specifically on critical incidents and patient experiences. After the workshops, questionnaires were emailed to participants, allowing them to elaborate on incidents or share additional ones. Phone interviews were conducted as an additional method to gain deeper insights into positive and negative experiences. Data collection was guided by a trigger statement designed to elicit critical incidents throughout the patient journey, focusing specifically on communication, information flow, and organisational aspects while deemphasising clinical factors such as illness, pain, and medication.

Participants were recruited through the Norwegian Cancer Society (NCS) and its district offices, targeting individuals impacted by cancer, including current and former patients and their next‐of‐kin. Recruitment varied across district offices: some distributed the information letter broadly, while others targeted specific individuals, typically active members deemed relevant to the study. A total of 67 individuals expressed interest. Some unable to attend workshops due to lacking digital skills or preferring not to participate in groups were offered phone interviews. Recruitment decisions balanced the need for sufficient data with manageable workshop sizes to ensure all participants could share their stories.

The final sample included 41 participants: 28 patients and 13 next‐of‐kin. Of these, 35 joined workshops, and six participated in interviews. Ages ranged from 31 to 79. Most patients had progressive cancer, while those in remission reported chronic conditions from cancer or its treatment. Most next‐of‐kin were spouses or partners. Data collection was conducted in May and June 2022, with written consent from all participants. Further demographic details are presented in Table 1.

**Table 1:** Demographic characteristics of the study participants (in total, *n* = 41).

Characteristics	Patients^a^ (*n* = 28)	Next‐of‐kin (*n* = 13)
Female	15	9
Male	13	4
Age range		
25–34	1	
35–44	1	3
45–54	4	3
55–65	8	2
65+	14	5
Cancer type		
Lung cancer	13	3
Prostate cancer	4	1
Breast cancer	3	3
Brain cancer	1	3
Other types: leukemia, intestine cancer, gynecological cancer, colorectal cancer, throat cancer, bone marrow cancer, esophageal cancer, unknown^b^	7	3

^a^ Five patients also had experiences as a next‐of‐kin.

^b^ One patient did not want to reveal the type of cancer.

### 2.2 Data Collection

**Figure 1:**
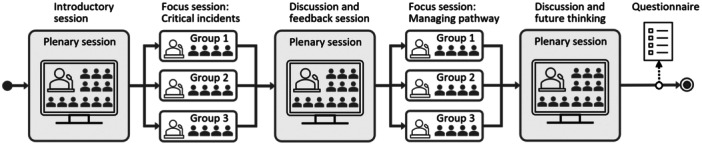
Workshop procedure for collecting patient experiences and critical incidents.

Data were collected from three workshops and six interviews conducted via Microsoft Teams. All sessions were recorded, with moderators also taking notes. Each two‐hour workshop alternated between plenary sessions and focus group sessions of 3–4 participants (see Figure 1). A detailed script guided the facilitator (Author 5) and group moderators (Authors 1, 2, 5) to ensure consistent data collection. After outlining participants' rights and the workshop agenda, an icebreaker exercise about patients' role in healthcare was conducted. A fictional patient journey was then introduced to familiarise participants with the concepts of “patient journey” and “actor”.

The first focus session was dedicated to collecting critical incidents. Following brief introductions by the participants, the moderator reiterated the session's topic and presented the trigger statement: “*Think of a specific episode where communication or information flow was particularly satisfying, perhaps exceeding your expectations (round two: dissatisfying, perhaps causing an extra burden)*.” After 3 min of individual reflection, each participant had 5 min to share their positive and negative episodes. Moderators encouraged uninterrupted sharing, offering supportive acknowledgements, while allowing brief reactions or affirmations from others. Following sharing, time was allocated for group discussion. Upon resuming the plenary session, the moderators summarised the incidents and gathered feedback. The second focus session addressed pathway management, and the final plenary explored future prospects. Questionnaires were emailed to all participants immediately after the workshops, with 23 out of 35 responding. The questionnaires included background questions but primarily aimed to gather incidents. Participants could elaborate on workshop incidents or describe new incidents.

Author 2 conducted six additional interviews, focusing on participants elaborating on particularly positive or negative episodes, similar to the first part of the workshops. Each interview lasted between 1 and 1.5 h. Data from the workshops, questionnaires, and interviews provided insight into participants' positive and negative experiences with communication and information flow during the cancer journey. Workshop accounts were briefer and less detailed than the more comprehensive episodes shared in the interviews due to time constraints.

### 2.3 Data Analysis

Data from the group sessions and interviews were transcribed with the moderators' notes as guides. All data, including questionnaire responses, were sorted into positive and negative patient episodes and compiled in an Excel spreadsheet. The analysis team (Authors 1–3) reviewed the episodes, and after an initial read‐through, established criteria for identifying critical incidents (CIs) based on [30]:

**C1. Critical:** Directly emerging from the trigger statement, excluding secondary or associative episodes.

**C2. Episodic:** Involves one or more actors in a discrete event with sufficient detail.

**C3. Scope:** Focuses on communication, information, and organisation.

An iterative analysis using criteria C1–C3 was conducted to extract CIs from the episodes. Multiple iterations and consensus meetings resolved conflicting classifications. Episodes varied in abstraction, ranging from detailed elaborations to brief descriptions, and spanned time periods from minutes to weeks. Longer episodes with multiple critical factors or new story transitions were divided into atomic units meeting the episodic criterion (C2). Duplicate episodes from the questionnaires were removed. In total, 304 episodes were collected, yielding 187 CIs after sorting and filtering (see Table 2).

**Table 2:** Patient episodes and the emerging critical incidents by source and positive/negative experience.

	Positive experiences		Negative experiences		All	
Data source	Patient episodes	Critical incidents	Patient episodes	Critical incidents	Patient episodes	Critical incidents
From workshops	64	**46**	90	**71**	154	**117**
From questionnaires	36	**25**	44	**24**	80	**49**
From interviews	34	**10**	36	**11**	70	**21**
Total count	134	**81**	170	**106**	304	**187**

*Note:* Bold values indicates counts for critical incidents.

**Figure 2:**
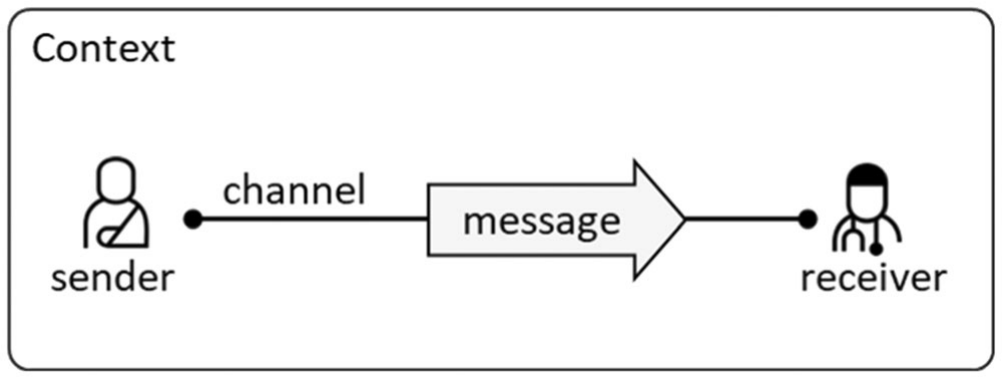
Semiotic model of communication, exemplified by a patient sending a message to a doctor.

While most CIT studies are qualitative, some quantitative studies exist [23]. Our analysis combined two approaches. First, a deductive approach quantified the actors and communication channels involved. CIs were analyzed using a semiotic communication model [31] (see Figure 2), where a *sender* transmits a *message* to a *receiver* through a specific *channel*, (e.g., speech or email) in a given *context*. This model enabled us to quantify the frequency of actors, channels, and whether messages reached their intended receivers (communication outcome).

The second approach used inductive content analysis [27] to examine CIs related to communication, information, and coordination in the healthcare system. This aimed to deepen understanding of the nature and antecedents of CIs while aligning with the semiotic model. The analysis involved iterative, careful readings of incidents to identify categories based on similarities. To ensure scientific rigor, two analysts (Authors 2 and 3) conducted the analysis. The categories were developed inductively, and consensus meetings were held to discuss and finalise the categories. The analysis resulted in 12 categories (see Section 3.2), reflecting aspects participants considered crucial to their perceptions of cancer care as positive or negative.

## 3 Results

A total of 187 critical incidents were identified from the interviews, workshops, and questionnaires, comprising 81 positive and 106 negative incidents. The average length was 150 words, with negative incidents being more detailed than positive ones. Incidents from interviews were typically longer, while those from questionnaires were shorter.

### 3.1 Overview of Actors, Channels, and Communication Outcomes

**Figure 3:**
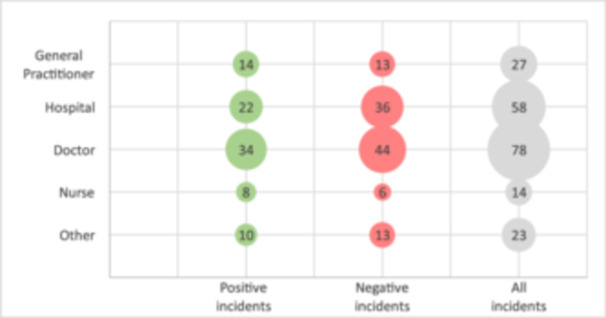
Frequency of various actors mentioned in critical incidents, illustrated using a bubble diagram. Note that multiple actors may be mentioned in a single incident.

Participants mentioned various actors in their critical incidents, including individual HCPs and institutions. The most frequently mentioned were general practitioners (GPs), hospitals, and hospital doctors and nurses. (In Norway, where the study was conducted, each patient has a dedicated personal GP). Other actors included cancer coordinators, the national health portal (Helsenorge.no), municipal health services, emergency care units, school health services, the labour and welfare administration, and patient transport services. Figure 3 shows the frequency of the most mentioned actors, while Table 3 provides illustrative examples.

**Table 3:** Excerpts from critical incidents highlighting various actors.

Actor	Positive incident	Negative incident
General practitioner (GP)	My GP has been a stable rock while everything else has been chaotic. Even though he is not necessarily very involved in the actual treatment process, he always makes time, clears his schedule, and even gave us his private number. We haven't used it, but just the fact that he offered it is reassuring.	My wife received the [bad] news (..) while we were at a shopping center buying Christmas presents. (..) It was positive that he [the GP] called and explained what would happen next in the treatment process, but what is terribly strange is that we haven't heard from him since. We were very surprised by that. The GP went completely silent and disappeared.
Hospital ‐ generic	When I had the setback, I was called in [to the hospital] along with my husband (..). In that conversation, we received a thorough review of the problem and the spread of the cancer, and what I could expect from the treatment going forward. (..) Since I had my husband with me, we managed to remember quite a bit. It was a new experience for me to receive such good information.	Before I was told that I had a serious illness, I was given an MRI report that described widespread cancer spread, without anyone talking to me about it or going through the report with me. So, I literally had to Google to find out that I had metastatic cancer.
Doctor	It was the doctor who both informed me that I had breast cancer and whom I also spoke with before and after the surgery. He was very, very supportive and explained everything very well. Unfortunately, he has now retired. They called him a “living Valium.” He was so calming; he was absolutely wonderful.	It started with a doctor from [hospital] calling and telling my wife: Your cancer is very serious. I don't know anyone who has survived, so you should expect this to be your last Christmas. He said this on December 15th. [She lived for 10 more years].
Nurse	The eldest of the cancer nurses took the initiative to have a conversation with me and my husband about sexuality after being treated for gynecological cancer. I know that many do not have this conversation and miss it. For us, this was very beneficial, and it is clear that this should be a topic for everyone.	My husband was admitted on a Thursday, and the nurse on duty said I could stay with him as long as I wanted. On Friday afternoon, however, a new nurse told me I could only stay two hours each day. Therefore, I went home after my allotted time on Saturday. When I returned on Sunday, I was told to inform our children that he was dying.
Other	The school health services have regularly talked with the children since my husband got sick. They have also informed them about the treatment my husband was going through, including reading books on the subject. The environmental therapist took them to a family park one day during their summer vacation so that the children could have a nice experience while their dad was hospitalized, and so I could have a day of respite.	The bad part was when the late effects started to appear, and I hadn't received any information about them. At the same time, it was a very tough situation because both of us were sick. I knew there was a cancer coordinator in the municipality, but I didn't have the strength to reach out, and she didn't contact me either—so that was disappointing.

**Figure 4:**
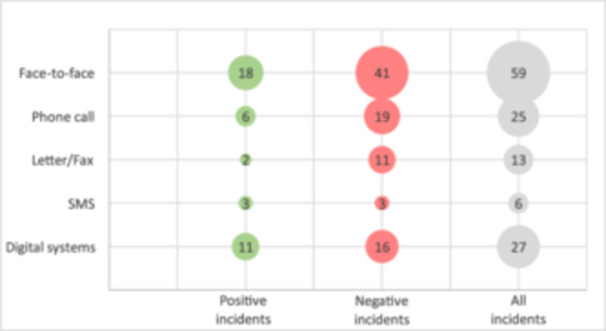
Frequency of various communication channels mentioned in critical incidents, illustrated using a bubble diagram.

About half of the incidents addressed the communication channel. Figure 4 shows the association between different channels and positive and negative experiences. Face‐to‐face communication, phone calls, and SMS were rarely central to the incidents, except when hospitals provided hotlines or HCPs shared personal mobile numbers for extended availability—both highly valued. In contrast, incidents involving letters, faxes, digital systems, and health portals often centred issues with the channel itself. Incidents involving letters or faxes often involved ad‐hoc strategies for incompatible systems, leading to delays. For digital systems, negative incidents stemmed from issues with hospital information exchange. Half of these cases involved patients receiving distressing diagnoses online before consulting a doctor. Positive incidents with digital systems highlighted the timely, high‐quality information available on health portals.

**Figure 5:**
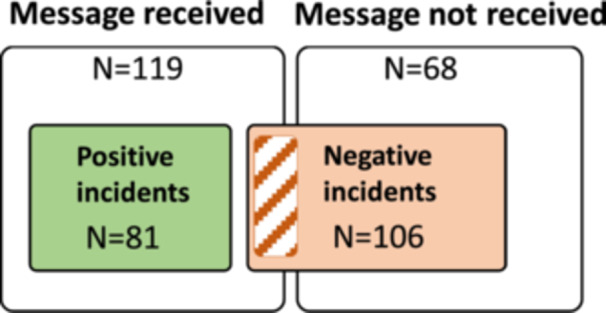
Venn diagram of communication outcomes in positive and negative incidents.

Using the semiotic model, we analyzed the “technical” outcome of communication—whether the message reached its intended recipient. In 119 incidents (64%), the message was successfully delivered, with most being positive (Figure 5). Negative incidents in this group often involved traumatic content or insensitive behaviour (Section 3.2.5). All 68 communication breakdowns (36%) were perceived negatively, consistent with the nature of the negative trigger statement. Table 4 provides example incidents for both communication outcomes.

**Table 4:** Excerpts from critical incidents concerning outcomes of communication.

Outcome	Positive incident	Negative incident
Message received	The chief doctor sent text messages in the evening and almost around the clock, which was very good. There was very open communication about my situation—both about what was good and what was not good.	When my father's condition significantly worsened, a doctor came into the room and said, “Well, well, now you're just going to get worse and worse.” (..) The other doctors we have met in the specialist healthcare service have been good and skilled. This was a negative experience that has left an impression and bothers me from time to time.
Message not received	n/a	They tried to send my CT scans from the local hospital to the regional hospital. This was not possible. (…) I received a CD (…) and had to drive to another local hospital which was able to send [the CD content] to the regional hospital so it was ready the next day when I arrived and inquired. Clearly, there is room for improvement.

In cases of communication failure, we assessed whether the patient had to take compensatory action. Lack of communication between actors in the patient journey was a common feature of negative incidents. In 41 of the 106 negative incidents (39%), patients acted as a messenger to ensure progress in their care.

### 3.2 Content Analysis of Critical Incidents

The content analysis identified 12 categories highlighting factors participants deemed critical to the experience of a good or a bad cancer journey. The categories (Table 5) are not mutually exclusive, and some topics may overlap.

**Table 5:** Categories identified in the content analysis.

Main area for critical incident	Actors involved	Category (number of critical incidents; positive/negative)	Meaning
Information and communication	Patient–healthcare	Access to information (14/16)	The patient has access to information when desired
		Timely information (1/15)	The information is available for the patient at the right time
		Information quality (17/5)	The information is understandable
		Informed healthcare workers (0/11)	Healthcare workers are informed about the patient's situation
		Clinical empathy (24/17)	Healthcare workers have empathy when interacting with the patient
		Patient and next‐of‐kin involvement (13/4)	Services apply a patient‐centred approach. Services involve next‐of‐kin
	Healthcare–healthcare	Communication and information exchange within the healthcare system (2/21)	Communication and information exchange within and across healthcare providers is satisfactory
Organisation	Patient–healthcare	Coordination (2/13)	Services are experienced as coordinated across healthcare actors
		Planned services (7/6)	The service is predictable
		Time and tempo (14/7)	The service is given in a speedy manner
		Access and availability to services (10/16)	Services exist and have capacity to help the patient
		One contact point (15/12)	Services provide patients with one contact point

#### 3.2.1 Access to Information

Most incidents in this category concerned communication and information exchange *between* the patient and healthcare. While access to information fosters positive experiences, lack of access led to negative ones. The wife of a man with aggressive cancer explained:

“After having waited for a month we were informed about the cancer diagnosis, we had to call the hospital because we did not know anything”

For many, lack of information caused stressful waiting periods and uncertainty. Positive experiences were tied to clear, timely and understandable information. One woman described: “Good communication flow, the GP has been very good, she informed me right away, the same day I had been to an MRI scan.”

#### 3.2.2 Timely Information

Timely information was deemed crucial during the cancer journey. However, few participants emphasised it in their positive incidents, with most associating it with negative experiences. Information was often provided too late or too early, and many patients reported learning about their diagnosis or progression through the health portal before hearing it from their physician:

“I had an MRI and PET scan of my head on a Tuesday. (..) In the evening, I was having dinner with my husband at the hotel, and we were enjoying ourselves. Then I got a message on the national health portal, concerning the exemption card. At the same time, I noticed a heading in my patient record. It was a document clearly stating I had stage 4 lung cancer.”

Such experiences were often described as dramatic. While not all incidents were equally intense, many participants reported receiving information too late or at an inappropriate time. For instance, one man was informed about medications immediately after anesthesia but only recalled the details when reading them on the health portal.

#### 3.2.3 Information Quality

Information quality was mostly described positively, with participants emphasising it as “clear,” “straightforward,” “open,” and “to‐the‐point,” particularly in oral communication. This often made it challenging to separate the quality of the information from the communicator. For instance, a female patient recalled receiving “clear information” about her radiotherapy and its necessity, while her husband and son were allowed into the control room, where they received a thorough explanation of the procedures. A lung cancer patient similarly shared:

“The communication between the outpatient clinic, the physicians, and me has been fantastic. Smooth flow of information, much patient involvement. Open about everything. I think it has been outstanding.”

#### 3.2.4 Informed Healthcare Workers

Another category within the information/communication area involved only negative incidents and concerned whether healthcare workers were informed about the patient's situation. Patients see many different providers, and only a few of them have access to the same health record. Consequently, patients' information must be transferred digitally via manual routines. Patients often noticed the lack of updates indirectly, whereas sometimes HCPs openly admitted that they were unprepared:

“The nurse responsible for my husband during chemo always opened the conversation and treatment by saying, ‘I haven't managed to update this time either’.”

A lack of information exchange between providers often forced patients to act as messengers to keep physicians and nurses updated. Although this was not a role patients wanted, many found it necessary and reassuring to take control of the information exchange. Some participants described creating systems, such as binders with printed EHR notes, laboratory reports, or custom Excel sheets and graphs, to track information during a patient journey.

#### 3.2.5 Clinical Empathy

The most frequent topic was HCPs' interpersonal skills and empathy toward patients and next‐of‐kin, linked to both positive and negative incidents. Positive incidents involved HCPs addressing patients' needs, seeing the “whole” person, and balancing clarity with empathy:

“The conversation [when she was told that she had incurable lung cancer] was a good one. I got the information I needed; the doctor was clear. She also gave me hope, while also being realistic (…) She was supportive; she met me in a good way.”

“My GP has been very good. There are so many instances where she sensed what we wanted to know.”

Positive interpersonal meetings were often followed by concrete actions, such as physicians and nurses making themselves accessible, sharing personal phone numbers, and taking responsibility for the patient.

Negative experiences stemmed from a perceived lack of empathy. Participants recalled abrupt cancer diagnoses that left them feeling hopeless, as well as later encounters where HCPs were “strict,” “angry,” and “unaffected.” Such experiences contributed to emotional and mental challenges:

“The first physician we met at the hospital gave my wife and me a very bad experience, offering no hope. She gave me a bad prognosis, having stage 4 lung cancer. She said, ‘There is nothing we can do here.’ (..) Especially for my wife, this became a horror experience. Now, five years later, after having immune therapy, we still talk about that episode – so it was pretty severe.”

#### 3.2.6 Patient and Next‐of‐Kin Involvement

This category includes incidents where HCPs placed patients and next‐of‐kin centre stage. Most experiences were positive, with few negative exceptions. Some participants shared general accounts of feeling taken seriously and well cared for. Others specifically mentioned communication and shared decision‐making:

“Confident around shared decisions.”

“[My doctor] chose to enter a dialogue with me – very nice – and didn't immediately say what he would recommend. It became a dialogue where I felt safe and cared for. I participated, and it made me feel in control.”

“We had a fantastic oncologist who called me almost every time a decision was made about my father's treatment (…) She kept us up to date and informed us about the medical assessments. I felt safe having this information directly from the doctor.”

Only two negative incidents were identified, and they were related to a lack of information and compassion for next‐of‐kin.

#### 3.2.7 Communication and Information Exchange Within the Healthcare System

This category covers “backstage” incidents where healthcare communicates or exchanges information without involving the patient directly. When communication breaks down, patients often face significant consequences, making this category almost entirely associated with negative incidents.

Patients often notice communication gaps when receiving care from both a local hospital and a regional university hospital. In Norway, specialised diagnostics and treatments occur at university hospitals, while local hospitals handle less advanced care. Many negative incidents arose from communication breakdowns between these institutions, though they also occurred between departments within the same hospital, as one participant shared:

“Poor communication between different departments during the diagnostic phase when they were trying to determine why I was feeling sick. My case was bounced back and forth between departments for half a year. Not good communication.”

Other examples of missing communication or ineffective information exchange forced patients and next‐of‐kin to act like messengers. One male patient was admitted to a hospital other than the one managing his cancer care. After undergoing CT scans during an acute admission, he requested the scans sent digitally to his regular hospital. When this proved impossible, he was given a CD to deliver personally. Although this incident occurred a few years ago, participants noted that they still occasionally act as messengers—a consistently negative experience.

Extensive specialisation in medicine, particularly in cancer care, has also contributed to negative incidents. Some participants felt overlooked as whole individuals with needs extending beyond cancer care, emphasising the importance of involving HCPs with broader competencies. One participant complained that her physician focused solely on her ovary and failed to inform other HCPs about her elevated blood values. She called for better communication and collaboration between specialists.

#### 3.2.8 Coordination

Healthcare coordination, closely related to the previous category, focuses on the organisational level and its ability to manage tasks and responsibilities effectively. Most incidents involved negative experiences, with a common issue being that patients and next‐of‐kin often had to assume the responsibility of coordinating services themselves. Coordination problems were reported both between and within hospitals.

One example of poor coordination between departments was shared by a daughter whose father was seriously ill. Due to his unstable condition, he was frequently hospitalised for infections caused by his cancer treatment. However, instead of being admitted to the cancer department, he was placed in the infection department. The lack of collaboration and coordination between these departments frustrated the daughter, eventually fearing her father's health:

“Messages from doctors were not followed up, and necessary measurements were neglected. Worse, he had a serious infection and was very ill; his O2 levels, temperature, and blood pressure were supposed to be monitored several times a day. This did not happen. I asked for it and brought it up several times.”

Another case illustrated how the healthcare financing can have unintended consequences. A man with prostate cancer shared that his local hospital was monitoring his condition. However, as Norwegian hospitals are organised into health trusts comprising multiple hospitals, he received simultaneous appointments from two hospitals within the same trust. His regular hospital staff explained that the financing system, based on preset fees, incentivizes maximising patient numbers. The participant found this practice disturbing.

#### 3.2.9 Planned Services

This category concerns the predictability of healthcare services. Careful planning and clear communication were generally viewed positively, while uncertainty and sudden changes were seen negatively. Positive and negative incidents were roughly equal in this category.

Several positive experiences were directly linked to CPPs, standardised care plans with predefined timelines for each step designed to make cancer diagnostics and treatment predictable. One patient praised this system:

“You know, when you're in the pathway, it's very good. It's like starting a computer program [that runs], ‘tok‐tok‐tok‐tok’ ‐ the first available appointment appears, and the first surgery slot is scheduled (…) it's a very well‐organized journey.”

Another example shows that planning and a predictable pathway are possible with simple measures. A woman talked about her husband:

“When he finished treatment, he's sent home with a written note listing the dates for new blood tests, video consultation, and the next treatment. It makes life much simpler, knowing what will happen six weeks in advance.”

Simple measures like these can greatly benefit patients and next‐of‐kin by providing clarity and reducing anxiety. Conversely, a lack of planning and unpredictable changes lead to negative experiences. Another wife shared a poor start to her husband's treatment:

“After being informed about the cancer and that it had metastasized, a ‘horrible waiting time’ followed. The hospital had scheduled a treatment start date, but it was canceled a few days before. He got a new appointment the following week, but also that was cancelled.”

Some negative incidents left it unclear whether the issue was poor planning or simply a lack of communication about otherwise good plans. Regardless, the outcome for the participants was the same: an unpredictable patient journey.

#### 3.2.10 Time and Tempo

In short, participants viewed rapid services as positive and slow services as negative, with more positive incidents reported. Positive examples included quick referrals to diagnostics, fast service delivery, and promptly receiving information, such as test results (cf. the category “Timely information”). Statements varied from “it went very quickly” to “the ambulance came instantly.” CPPs were highlighted in this context for facilitating rapid patient journeys, according to the participants:

“Everything has been very good. I was included in a cancer patient pathway, and it moved quickly. (…) Diagnostics and treatment were immediately put into action, and it went very quickly.”

Negative incidents in this category were fewer and mostly reflected the opposite of positive ones, such as long waiting times and delayed treatment. One patient waited 1.5 months for a referral from the local hospital to the university hospital, followed by another 1.5‐month wait after diagnosis. Although enrolled in a CPP, he was told that the target times were guidelines, not legally binding. He found it difficult, saying “You know you have cancer, but nothing more.”

Another patient highlighted a different issue—insufficient time. He felt that his hospital visits were too brief to receive thorough information or ask questions.

#### 3.2.11 Access and Availability of Services

Cancer diagnostics and treatment are primarily provided by specialist health services, with participants generally reporting good access. However, most critical incidents involved access to primary care, including cancer care coordinators and rehabilitation, with more negative than positive incidents reported.

Positive incidents included access to a 24/7 emergency hotline with minimal wait times to reach an oncologist and a municipal cancer nurse providing home visits. Participants also highlighted a municipal cancer coordinator and a GP who was “always” available as critical positive experiences.

Negative incidents included missing services, such as the absence of cancer care coordinators in some municipalities, leaving patients without crucial support. Others mentioned that even when these services existed, they did not reach out to them, and patients were often too ill to seek help themselves. One participant expressed the need for someone in the community to organise and explain their situation, as he and his wife struggled to understand the details and treatment options provided by HCPs. Another patient mentioned the lack of follow‐up, including the absence of someone to talk to, as a negative critical incident:

“In retrospect, I think it would have been very good to have had someone to talk to – not just about the cancer, but about the whole situation, how I felt. I was never offered anything like that (…) I had no one to talk to about anything other [than the cancer illness].”

#### 3.2.12 One Contact Point

Many participants highlighted the importance of a single contact point for continuity of care, considering it invaluable. Conversely, the lack of such contact was frequently as negative. Having a GP, hospital doctor, or cancer coordinator who was always available and supportive was considered critical.

“In the beginning, there were many physicians involved, but once I had one dedicated physician in the cancer ward, it worked really well.”

Negative incidents often stemmed from frequent staff changes, causing patients to constantly encounter new physicians and nurses:

“He's had a new doctor every time we came to an appointment. (…) Since 2018, I don't think I've ever had consecutive conversation with the same person. They are all nice individually, but it's unfortunate when they don't have the history that matters.”

The lack of a single contact point, combined with frequent staff rotations and limited time to review patients' histories, often left patients unsure about the adequacy of their care and treatment.

## 4 Discussion

In this study, we used CIT to examine pivotal patient experiences in the cancer journey, focusing on communication, information, and care coordination. Using multiple data collection methods, we identified a range of positive and negative incidents, organised into twelve categories through content analysis. The journey perspective is increasingly recognised for understanding end‐user experiences [28, 32, 33]. CIT is well‐suited for retrospective studies, effectively capturing critical experiences from individuals' lived experiences [25, 26]. We actively primed participants with the journey concept before presenting the trigger statement to reduce recall bias associated with the lengthy and complex nature of cancer journeys. As one patient, whose journey spanned 19 years, reflected: “The positive episodes outnumber the negative ones, but the negative ones are more deeply embedded in my memory.”

Consistent with our findings, a systematic review highlighted the critical role of care coordination in determining cancer care quality in the USA, United Kingdom, and Canada [7]. A study from England identified care administration and coordination as the strongest predictors of patient satisfaction [34]. Similarly, our study revealed several negative incidents linked to poor care coordination. While positive incidents often related to time, tempo, and having a single point of contact—key aspects addressed by the CPP—participants also reported challenges with access and service availability. Many emphasised the importance of being treated with respect and dignity by HCPs, echoing previous research that underscores clinical empathy as crucial in the physician‐patient relationship [35].

Access, timeliness, and information quality emerged as key categories in our content analysis, with the timing of information being a frequent concern. A network analysis from England found that timely information improves patient satisfaction [17]. Participants reported negative experiences with both timing and communication among HCPs. In several cases, sensitive information intended for HCPs was prematurely disclosed to patients through digital patient portals. As these systems become more common, future studies should examine their impact and ensure they meet individual patient needs.

This study found that nearly 40% of negative incidents were linked to fragmented health services and poor coordination of care, often leaving patients to act as messengers. The involvement of multiple healthcare levels and providers increases the risk of fragmented journeys, complicating coordination and information flow [6]. As care complexity grows, positive care experiences are affected. Service research highlights the integrative role of customers in connecting service providers, a role mirrored in healthcare, where patients often connect HCPs involved in their care [36]. Various tools have been proposed to enhance cancer care coordination and communication [37]. Meanwhile, the dynamics of cancer care are shifting with precision medicine [1, 2, 38, 39], rapid adoption of digital technologies [40], and the shift to remote care accelerated by the COVID‐19 pandemic [40, 41]. These changes underscore the need for improved care integration models. A recent review [9] identified process management and integrated service delivery as key strategies to enhance efficiency and QoC.

### 4.1 Strength and Limitations

The multi‐method approach used in this study allowed us to enhance the efficiency and effectiveness of retrospectively identifying critical incidents. Cancer care quality can be studied from various stakeholder and system perspectives, but this study's novelty lies in adopting a patient journey perspective. By using strong priming techniques, we successfully elicited detailed recollections from patients and their next‐of‐kin. Patients' perspectives are crucial in cancer care due to its chronicity and overwhelming toll of the disease on the patients and their next‐of‐kin [8, 42]. The method, which included a warm‐up exercise, proved effective in gathering detailed patient episodes focused on critical incidents throughout the patient journey.

In this study we employed various methods to collect critical incidents from patients and next‐of‐kin. We found that the critical incidents collected through workshops were more complete than from questionnaire, which is in line with previous findings [7, 43]. Questionnaires could work but would require careful priming and explanation. Our recruitment aimed to include diverse patient groups and cancer types nationwide. However, recruiting participants from a patient organisation may limit the representativeness of the sample, as these individuals may be more engaged, informed, or proactive compared to the general patient population. Being an exploratory study, the relatively small sample size inherently limits the generalisability of quantitative findings. Due to COVID‐19, virtual workshops were conducted, restricting participation to digitally competent individuals. On the other hand, this enabled nationwide participation and geographical diversity.

## 5 Conclusion

Understanding patients' information needs and perceptions of care coordination is essential for optimising cancer care, and the workshop technique proved highly effective for examining critical experiences. This study highlights key areas for improvement, including delivering timely information and enhancing coordination among HCPs. On the human side, clinical empathy is crucial when delivering traumatic content such as cancer diagnosis. On the technical side, safeguards are necessary to prevent patient diagnoses from accidental or premature disclosure through health portals.

## Author Contributions

**Ragnhild Halvorsrud:** funding acquisition, writing – original draft, methodology, validation, visualisation, conceptualisation, investigation, writing – review and editing, project administration, data curation. **Line Melby:** writing – original draft, funding acquisition, methodology, validation, conceptualisation, investigation, writing – review and editing, data curation. **Kristine Gjermestad:** writing – original draft, methodology, validation, conceptualisation, investigation, writing – review and editing, data curation. **Binyam Bogale:** validation, investigation, writing – review and editing, conceptualisation. **Ingrid Konstanse Ledel Solem:** writing – original draft, methodology, validation, conceptualisation, investigation, writing – review and editing.

## Ethics Statement

Ethical approval was granted by SIKT—Norwegian Agency for Shared Services in Education and Research (Reference No. 248882).

## Consent

All participants, including people with cancer and their next‐of‐kin, provided written informed consent.

## Conflicts of Interest

The authors declare no conflicts of interest.

## Data Availability

The authors have nothing to report.
